# The Ability of Paramedics to Accurately Locate Correct Anatomical Sites for Intraosseous Needle Insertion

**DOI:** 10.7759/cureus.33355

**Published:** 2023-01-04

**Authors:** Daniel Berger, Alexandra Petrie, Jeffrey S Lubin

**Affiliations:** 1 Department of Emergency Medicine, Penn State Health Milton S. Hershey Medical Center, Hershey, USA; 2 Department of Obstetrics and Gynecology, Medical College of Wisconsin Affiliated Hospitals, Milwaukee, USA; 3 Department of Public Health Sciences, Penn State College of Medicine, Hershey, USA

**Keywords:** paramedic, anatomic location, ems education, intraosseous drill, emergency medical service, intraosseous infusion

## Abstract

Introduction

Intraosseous (IO) access is an alternative to peripheral intravenous access, in which a needle is inserted through the cortical bone into the medullary space using either a manual driver or an electric drill. Although studies report high success rates of IO access, failures are often attributed to incorrect site placement due to failure to adhere to anatomical landmarks. This study was designed to evaluate the ability of paramedics to locate the correct anatomic location for IO needle insertion.

Methods

Participants were paramedics who were recruited at Pennsylvania’s annual statewide Emergency Medical Services (EMS) conference. After completing a demographics survey which included information about their training and practice environment, they were asked to identify which IO sites were permitted for IO placement using the EZ IO® drill and to place a sticker at those locations on a human volunteer. A transfer sheet was utilized, and the distance between the participants' sticker and the location as marked by a physician board-certified in both Emergency Medicine and Emergency Medical Services was recorded. Descriptive statistics and t-tests were calculated from the records.

Results

Of 30 paramedics who participated in the study, 25 (83%) had been in practice for more than five years (range: 1-37 years), 13 (46%) reported running more than 20 calls per week, and 23 (79%) reported that they only or mostly provide 9-1-1 EMS response. Ten (36%) participants were currently certified in PHTLS, and 16 (57%) had previously been PHTLS certified. All participants reported having been trained in IO insertion. Seventeen (57%) reported having utilized an IO ≤10 times in the field, and 13 (43%) reported >10 field IO insertions.

When asked to identify appropriate IO insertion sites for the EZ IO drill, 26 paramedics (90%) correctly identified both the proximal humerus and proximal tibia. The average distance from the landmark for the humeral insertion site was 5.06 cm, with a statistically significant difference in the means for those who did and did not rotate the arm internally before identifying the humeral IO insertion site (*p *< .01). The average distance from the landmark at the tibial insertion site was 4.13 cm.

Conclusion

Although a high percentage of paramedics were able to verbally identify the correct location for IO placement, fewer were able to locate the insertion site on a human volunteer. Our results suggest a need for hands-on refresher training to maintain competency at IO insertion, as it is a rarely utilized procedure in the field.

## Introduction

Intraosseous (IO) access is an alternative to peripheral intravenous access (PIV), in which a needle is inserted through the cortical bone into the medullary space using either a manual driver or an electric drill [1]. Unlike vessels, the medullary space does not collapse during periods of hypotension, providing an opportunity to administer medications and fluids and obtain lab work when a PIV is not quickly or easily obtainable. IOs have been shown to have higher insertion success rates than PIVs during resuscitative thoracotomy, and the 2020 American Heart Association Guidelines for Cardiopulmonary Resuscitation and Emergency Cardiovascular Care affirm IO access as an alternative if PIV attempts are unsuccessful or not feasible [2,3].

Multiple IO insertion sites can be accessed with different IO needles, including the sternum, humerus, distal femur, proximal tibia, and distal tibia [1]. Studies report high levels of success with IO insertions, often describing success rates of greater than 90% for IO drills and greater than 70% for manual IOs [4-6]. Studies investigating IO access in both adult and pediatric patients often identify incorrect site placement or failure to adhere to anatomical landmarks as reasons that IO attempts were unsuccessful [5,7-8].

We hypothesized that paramedics would have a low level of accuracy in locating the correct anatomic location for IO needle insertion on a volunteer.

## Materials and methods

Our study was a prospective observational study to assess the ability of paramedics to properly identify the correct locations for both proximal humeral and proximal tibial IO insertion sites. This study was reviewed and approved by our Institutional Review Board.

We recruited study subjects over a two-day period at Pennsylvania’s annual statewide Emergency Medical Services (EMS) conference. After obtaining consent, participants completed a form with demographic data, including EMS practice information (number of years of EMS experience, area of practice, calls per week, and percentage of EMS response vs. patient transport) and certification in Prehospital Trauma Life Support (PHTLS), for each participant. We also asked participants whether they had previously received training specific to use of IOs and how many times they had inserted IOs in the field.

We utilized the Arrow® EZ IO® Intraosseous Vascular Access System (Teleflex Incorporated, Wayne, USA) for this study. We were attempting to measure two outcomes: paramedic knowledge of where the EZ IO® could be placed and the ability of the paramedics to locate two of the most commonly used locations on a real person. To demonstrate knowledge of proper sites for placement, participants were asked to identify which IO insertion sites (proximal tibia, distal tibia, sternum, clavicle, proximal humerus, or distal humerus) were permitted for IO placement using the EZ IO® drill, per the manufacturer. To evaluate the ability of the participants to locate the correct placement of the EZ IO®, participants were asked to place a sticker directly on the volunteer where they would insert the EZ IO® at both the proximal tibial and humeral insertion sites. Before recruiting participants, a physician board-certified in both Emergency Medicine and Emergency Medical Services created a template by identifying the correct IO proximal tibia and humeral placement sites on two male volunteers with normal body mass indices (BMI). The volunteers were shirtless and wore shorts to ensure participants could palpate landmarks. A transfer sheet was then applied to the volunteer to record the locations of the stickers. The template was placed over each participant’s sheet, and the distance between the two points was measured to the nearest 0.5 centimeters. A researcher recorded whether the arm was internally rotated to protect the biceps tendon before humeral insertion.

Descriptive statistics were compiled from the survey data and measurements. Percentages of paramedic characteristics are based on the number of participants who answered the question. A t-test was utilized to compare the mean distance of the identified humeral IO sites for participants who did and did not rotate the humerus.

## Results

Of the 30 paramedics who participated in the study, 25 (83%) had been in practice for more than five years (range: 1-37 years), 13 (46%) reported running more than 20 calls per week, and 23 (79%) reported that they only or mostly provide 9-1-1 EMS response. Ten (36%) participants were currently certified in PHTLS, and 16 (57%) had previously been PHTLS certified. All participants reported having been trained in IO insertion. Seventeen (57%) reported having utilized an IO ≤10 times in the field, and 13 (43%) reported >10 field IO insertions. Complete demographic and EMS practice information is shown in Table 1.

**Table 1:** Demographics of Participants *Values may not sum to 30 due to missing data. Percentages are calculated using total respondents as a denominator. **Respondents could select multiple options.

Years of EMS Practice	
1 to 5	5 (16.7%)
6 to 10	3 (10.0%)
11 to 20	9 (30.0%)
>20	13 (43.3%)
Calls per Week*	
0 to 20	15 (53.6%)
>20	13 (46.4%)
EMS Practice Type*	
All EMS	11 (37.9%)
Mostly EMS	12 (41.4%)
Mostly Transport	1 (3.4%)
Equal	5 (17.2%)
EMS Practice Area**	
Rural	21 (72.4%)
Suburban	1 (3.4%)
Urban	15 (51.7%)
Air	3 (10.3%)
IO Training	
Yes	30 (100.0%)
PHTLS Certification*	
Current	10 (35.7%)
Previous	6 (21.4%)
Never	12 (42.9%)
Estimated # IO Insertions Performed in the Field	
0 to 10	17 (56.7%)
>10	13 (43.3%)

When asked to identify appropriate IO insertion sites for the EZ IO drill, 26 paramedics (90%) correctly identified both the proximal humerus and proximal tibia. Twelve of those paramedics (41% of the total) also correctly identified the distal tibia as an acceptable location. Four paramedics incorrectly identified the sternum as an acceptable insertion site, and one also incorrectly identified the distal humerus.

**Figure 1:**
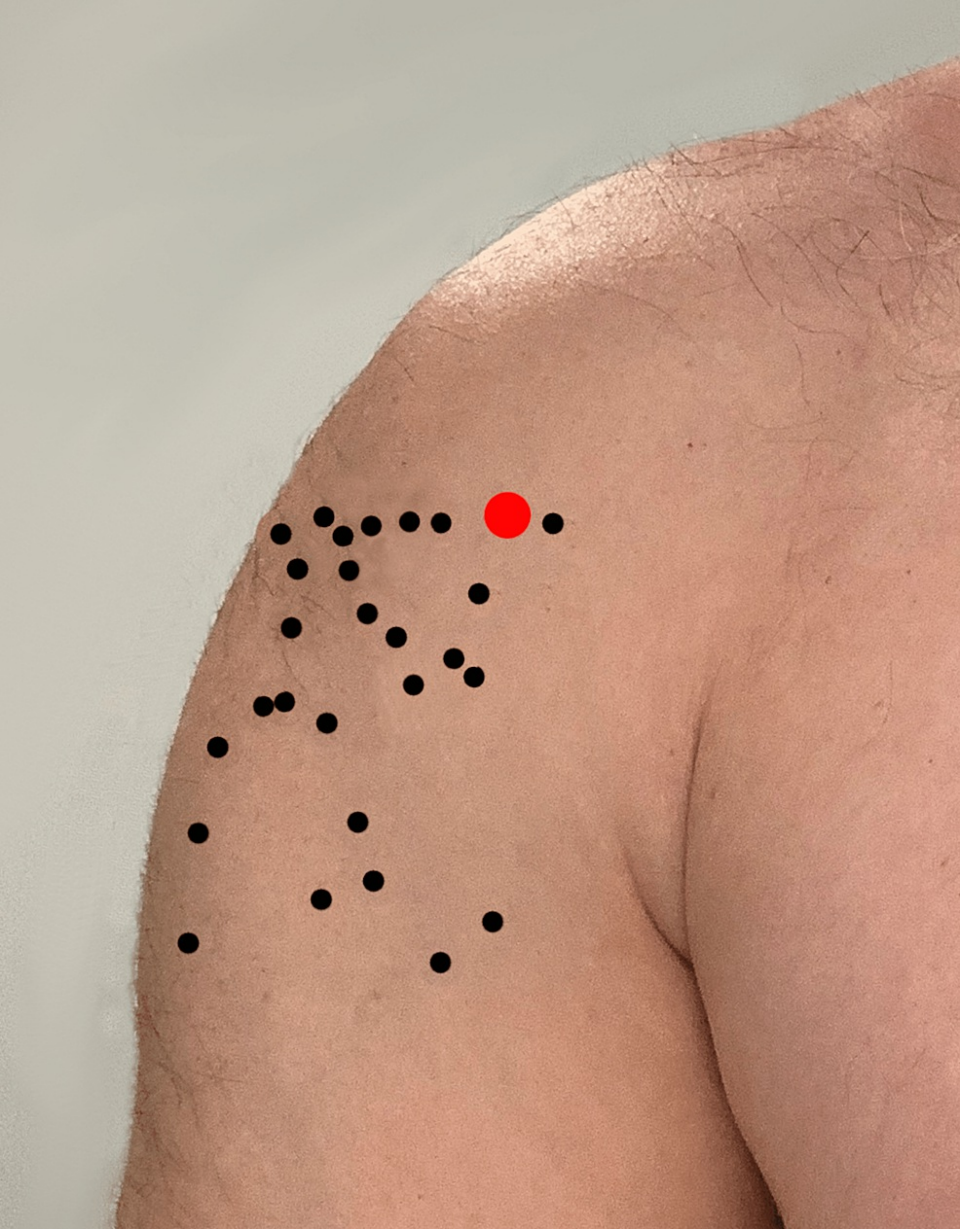
Diagram showing sticker placement by EMS providers (black dots) compared to the target placement (red dot) of humeral EZ IO needle

When asked to identify the insertion sites on a live volunteer, the average distance from the landmark for the humeral insertion site was 5.06 cm. The locations of proposed insertion sites are pictured in Figure 1.

Eleven (37%) paramedics correctly rotated the arm internally before identifying the humeral IO insertion site. When we analyzed data separately for those who internally rotated the humerus before placement and those who did not, the average distances from the landmark were 3.32 cm (standard deviation [SD] 2.41 cm) and 6.07 cm (SD 2.32 cm), respectively. The difference in mean distances was statistically significant (p < .01).

**Figure 2:**
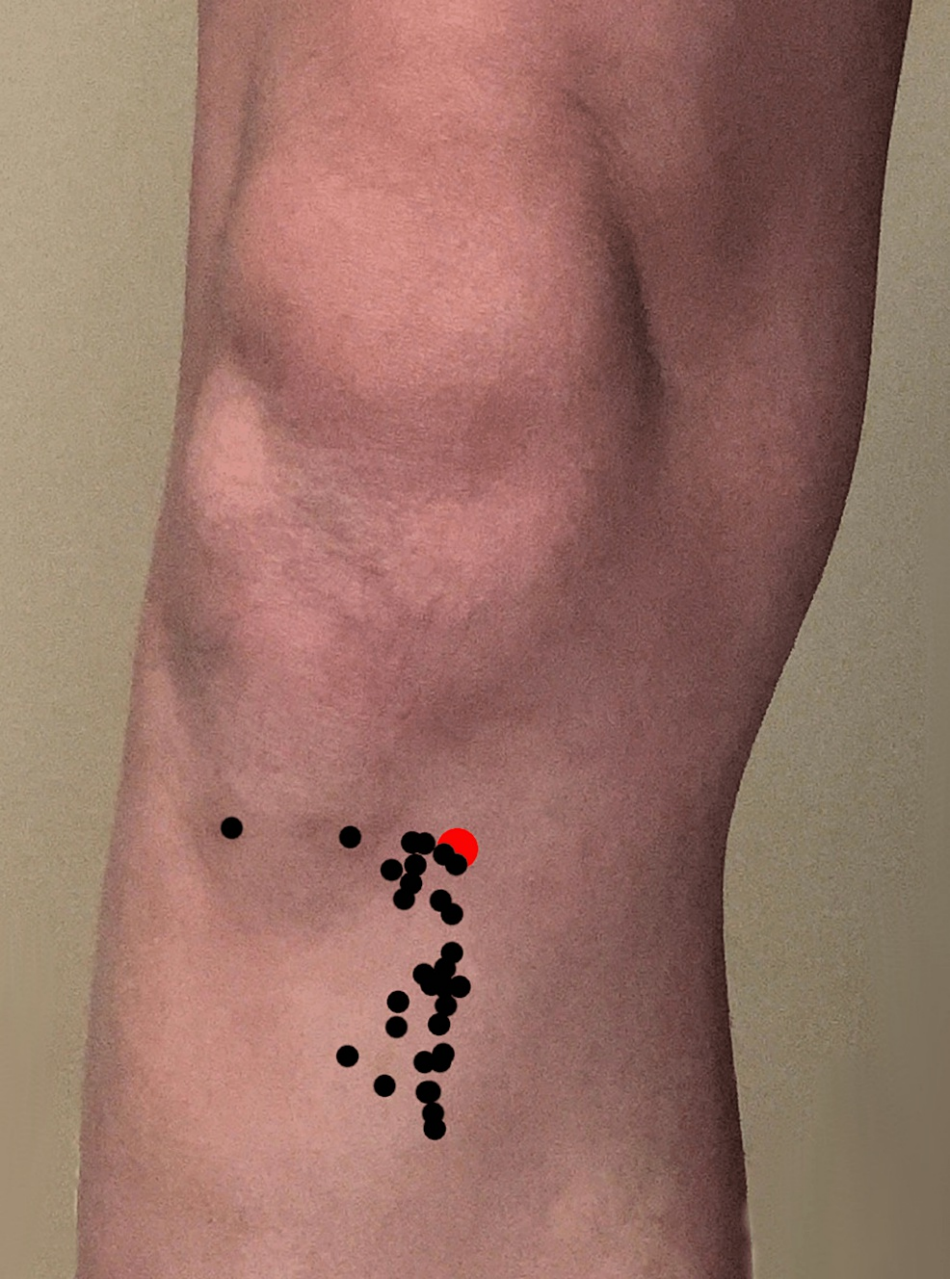
Diagram showing sticker placement by EMS providers (black dots) compared to the target placement (red dot) of tibial EZ IO needle.

The average distance from the landmark at the tibial insertion site was 4.13 cm (SD 2.59 cm), with proposed insertion sites located as pictured in Figure 2.

The distribution of distances is shown in Table 2.

**Table 2:** Distribution of the distance of IO insertion sites from the correct insertion site selected by participants.

Distance from Correct Insertion Site (cm)	Humeral n (%)	Tibial n (%)
0	1 (3.3%)	2 (6.7%)
0.25 - 2	3 (10.0%)	7 (23.3%)
2.25 - 3	4 (13.3%)	2 (6.7%)
3.25 - 5	6 (20.0%)	8 (26.7%)
5.25 - 7	10 (33.3%)	5 (16.7%)
7.25 - 9	4 (13.3%)	6 (20.0%)
>9.25	2 (6.7%)	0 (0.0%)

## Discussion

While most of the paramedics were verbally able to correctly identify the EZ IO® insertion sites, many were unsuccessful in correctly locating the insertion sites on the live volunteer. Participants appeared to have more difficulty locating the humeral insertion site than the tibial site. Previous literature of real-world and cadaver-based studies has reported higher success rates for tibial IO insertions than humeral insertions, with humeral insertion failures often resulting from participants incorrectly locating the humeral head [9-10]. The difficulty with landmark identification may also have been present our study, as only 37% percent of participants internally rotated the arm prior to locating the correct humeral insertion site.

We were unable to locate previous studies which identified the maximum distance from the landmarks that an IO can be placed and still be viable in a live patient. However, based on measurements of 195 tibias, Slocum et al. recommended that for IO access to be successful, the insertion site should be placed within the middle 50% of the width of the medial face and between 1.2 and 5 centimeters from the apex of the tibial tubercle [11]. In this study, we only recorded the distance, not the direction, from the correct insertion site. However, 70% of participants selected a site >2 cm from the physician-identified location.

Previous research has found that novices can be quickly trained to obtain IO access. In a study of Emergency Medicine attendings, residents, non-emergency physicians, and paramedics who were provided a five-minute training and demonstration utilizing the EZ IO®, although none had previously used the EZ IO® and more than 80% had never inserted an IO, first attempt success rate was 96.9% [12]. A similar study involving a group of emergency and non-emergency physicians, paramedics, and other healthcare staff who received an approximately one-hour lecture and were then assigned to utilize a manual intraosseous infusion system (Cook Medical Inc., Bloomington, USA) or the EZ IO® reported that although 84% percent of participants had no prior experience inserting an IO,79.5% had first-time success rates while using the manual needle and 97.8% using the EZ IO® [4]. A third study investigated the ability to train Emergency Medical Technician-Basic (EMT-B) students who do not have IO access within their scope of practice on correct sternal IO placement. After receiving a two-hour course on utilization of the First Access for Shock and Trauma (FAST-1) (Pyng Medical Corporation, Vancouver, Canada), 96.6% of participants successfully palpated the sternal notch IO landmark on a manikin [13].

While these studies show that novices can successfully place an IO immediately after being taught, IO insertion is an infrequently utilized skill. In our study, less than half of the paramedics reported that they had used an IO more than 10 times in the field, despite an average of 18 years of practice. This suggests that the ability to locate the correct anatomical locations for IO insertion on a live person may diminish with lack of hands-on practice.

We believe that our study is the first to investigate differences between paramedics’ ability to verbalize the correct location for IO insertion and to locate the correct IO insertion site on a live volunteer. However, other studies have found similar results regarding needle thoracostomy (NT). Ferrie et al. found that out of a group of 25 emergency physicians, 84% of whom were Advanced Trauma and Life Support certified, 88% were able to verbalize the correct landmark for NT, but only 60% were able to correctly locate that site on a volunteer [14]. Lubin et al. found that when asked to verbalize the correct location for needle decompression, 72% of paramedics identified the second intercostal space and 31% also noted that the insertion site should be at the midclavicular line [15]. Seventy-nine percent also correctly identified an alternate insertion site. However, when asked to mark the insertion site on a volunteer, only 28% identified a location within two centimeters of the correct anterior insertion site. These studies suggest that there is a disconnect between the ability to verbalize the “textbook answer” and the ability to locate the correct anatomical site.

Limitations

Our study results are limited by a small sample size and a convenience sample of Pennsylvania paramedics who attended the statewide EMS conference. It is possible that paramedics in different settings may use IOs more regularly or have more hands-on refresher training. Additionally, the questionnaire that the paramedics completed before IO insertion did not include questions about whether they had received additional IO practice with a cadaver or mannikin, whether IO attempts in the field were successful, and how long it had been since they were last trained on IO utilization, all of which could be confounding variables. Differences in initial IO training, including potentially incorrect technique, also had the potential to affect the study results. Furthermore, our “correct” insertion sites were identified by a single physician and were not verified with imaging, so it is theoretically possible that the site utilized for the template could have been improperly marked. The use of two different volunteers, even with similar anatomy, potentially could have affected results. Due to the small sample size, statistical comparisons between the distances marked on the two volunteers, or the various participant demographics, were not feasible.

## Conclusions

Although a high percentage of paramedics in our study were able to verbally identify the correct location for IO placement, few were able to locate the insertion site on a human volunteer. Since existing literature demonstrates a high IO insertion success rate for novices after only minimal training, it is reasonable to believe the paramedics in this study would have been capable of locating the correct IO site on a patient. However, the low number of field IO insertions relative to years of paramedic practice among our study population indicates that this is a rarely used skill. Our results suggest a need for hands-on refresher training to maintain competency at IO insertion and potentially other rarely utilized procedures.
